# Predicting the Remaining Lifespan and Cultivation-Related Loss of Osteogenic Capacity of Bone Marrow Multipotential Stromal Cells Applicable across a Broad Donor Age Range

**DOI:** 10.1155/2017/6129596

**Published:** 2017-02-19

**Authors:** Sarah M. Churchman, Sally A. Boxall, Dennis McGonagle, Elena A. Jones

**Affiliations:** ^1^Leeds Institute of Rheumatic and Musculoskeletal Medicine, University of Leeds, Beckett Street, Leeds LS9 7TF, UK; ^2^NIHR Research Unit, Chapel Allerton Hospital, Leeds Teaching Hospital NHS Trust, Chapeltown Road, Leeds LS7 4SA, UK; ^3^School of Molecular and Cellular Biology, Faculty of Biological Sciences, University of Leeds, Leeds LS2 9JT, UK

## Abstract

*Background and Objectives*. Culture expanded multipotential stromal cells (MSCs) have considerable potential for bone regeneration therapy but their wider use is constrained by the lack of simple and predictive assays of functional potency. Extended passaging leads to loss of multipotency but speed of decline depends on MSC donor age. The aim of this study was to develop an assay predictive of MSC culture longevity applicable to a broad donor age range.* Materials and Methods*. Bone marrow (BM, *n* = 7) was obtained from a diverse range (2–72 years) of healthy donors. MSCs were culture expanded to senescence and their osteoprogenitor content, gene expression profiles, epigenetic signature, and telomere behaviour were measured throughout. Output data was combined for modelling purposes.* Results*. Regardless of donor age, cultures' osteoprogenitor content correlated better with remaining lifespan (population doublings before senescence, PD-BS) than proliferative history (accrued PDs). Individual gene's expression or telomere length did not predict PD-BS but methylation of individual CpG islands did, PRAMEF2 in particular (*r* = 0.775). Coupling the steep relationship of relative* SPARC* expression with PD-BS (*r* = −0.753) the formula* SPARC* × 1/PREMEF2 gave an improved correlation (*r* = −0.893).* Conclusion*. A formula based on* SPARC *mRNA and PRAMEF2 methylation may be used to predict remaining BM-MSC longevity and related loss of multipotentiality independent of donor age.

## 1. Introduction

Bone marrow (BM) multipotential stromal cells (MSCs), also termed skeletal stem cells [1, 2] or mesenchymal stromal cells [3], have considerable potential for bone regeneration. To date, preclinical and clinical studies have been conducted using both culture expanded and noncultured BM MSCs and showed promising, but often variable, clinical results [4]. This could be due to the current lack of robust quality control assays to determine cultures' functional potencies prior to implantation.

Batch testing of MSCs for their purity control is established based on cell surface markers [5]. Screening of an individual batch's longevity and differentiation capacity is generally not performed by MSC manufacturers but is important to consider in order to ensure consistent clinical results. The number of cells needed for transplantation is the key issue in the field, particularly for very large bone defects, and is the reason why significant culture expansion is in many cases unavoidable; however it is known to lead to a gradual loss of multipotentiality and an increase in the proportion of senescent cells [6] (a phenomenon termed “MSC replicative ageing”) [7]. It is common for MSCs to be introduced to a patient following approximately 3 passages in culture but this in itself is not a precise measure of replicative ageing as cumulative population doublings (PDs) are dependent upon the initial numbers of seeded MSCs. Furthermore, cumulative PDs at passage 3 (p3) are unlikely to be indicative of the culture's remaining lifespan, which is related to both cumulative PDs and donor age [8–10].

The aim of this study was to develop a predictive test for the remaining lifespan and functionality of cultured BM MSCs based on the dynamics of gene expression alone or in combination with other parameters [11], which would be applicable throughout culture expansion stages and valid regardless of donor age. The DNA-methylation status of six CpG sites was investigated as a representation of the proximity of cellular senescence in relation to the stage of in vitro culture [12]. Gene expression and CpG methylation levels were combined in order to propose the most predictive model.

## 2. Materials and Methods

### 2.1. Generation of MSC Cultures

Iliac crest BM aspirates were obtained from 7 healthy donors following informed written consent (mean donor age 28.4 years; male 2, 20, 22, 22, and 72 years, female 19, 35 years). Ethical approval (06/Q1206/127 and 07/Q1205/27) was obtained from the National Research Ethics Committee (Yorkshire and Humberside).

MSC cultures were established from 100 *μ*L of BM aspirate seeded into 10 cm dishes and expanded in Alpha-MEM (Life Technologies, Paisley, UK) containing 10% FBS (PAA, Little Chalfont, UK) and 1 ng/mL FGF2 (PeproTech, London, UK) [13]. Three donor marrows (22, 35, and 72 years) were also expanded in StemMACS medium (Miltenyi Biotec, Bisley, UK). Cultures were maintained with twice weekly half media changes. At day 21, population doublings (PDs) were calculated using following equation: PD = log⁡2^(cell count on day 21/CFU-F on day 0)^; this culture was defined as p0. CFU-Fs (colony-forming units-fibroblast) on day 0 were determined from two further replicate dishes that were enumerated on day 14 following Crystal Violet staining; one colony was defined as >50 cells [14]. The remainder of the samples were passaged at the cell seeding density of 4500/cm^2^ and expanded to senescence, defined as less than 1 PD achieved in 7 days (presenescent being the previous passage) [15]. BM-MSC cultures were established and maintained by two individuals (SC and SB) who worked exclusively with each culture.

### 2.2. Flow Cytometry

To prove cultures' MSC identity p1 and p3 cultures were trypsinised and stained with the following antibodies: CD90-PE, CD105-PE, CD31-FITC (all from Serotec, Kidlington, UK), CD73-PE, CD34-PerCp, CD45-PE-Cy7, CD19-FITC, CD14-PE, CD146-PE, and HLA-DR-FITC (all from BD Biosciences, Oxford, UK). All antibodies were used at manufacturers' recommended concentrations and the data were collected and analysed using a LSRII flow cytometer equipped with FACSDiva Software (both from BD Biosciences).

### 2.3. The Assessment of MSC Osteogenic Capacity at the Clonal Level

Colony-forming unit-osteoblast (CFU-O) assays were performed with culture expanded cells at regular intervals throughout their culture period. For each assay, 500 MSCs were seeded into 6 replicate 3 cm dishes in media as described above; on day 8 colonies were washed in PBS and the medium changed to standard osteogenic medium [16]. Half media changes were carried out twice weekly. After 14 days, alkaline phosphatase activity assays were performed (*n* = 2 dishes), and after 21 days calcium production assays (*n* = 3 dishes) were carried out and calcium levels were determined following HCL extraction using calcium liquid (Sentinel Diagnostics, Milan, Italy) [16]. Osteogenically differentiated MSCs from the 6th dish were used for qPCR at day 21.

### 2.4. Quantitative Real-Time PCR

Aliquots of culture expanded MSCs were lysed during passage and oMSCs directly in the dish using lysis buffer from an RNA/DNA/protein kit (Norgen Biotek, Thorold, Canada). RNA and genomic (g) DNA were used in this study. RNA from senescent cells was not used due to degradation. RNA was reverse transcribed to cDNA using High Capacity reverse transcription kit and gene expression was measured by quantitative real-time PCR (qPCR) using a 96-gene Custom Taqman Array (both from Life Technologies) [17]. Genes were selected to encompass the multipotential nature of MSCs with particular emphasis on the bone lineage since the focus of this study was their osteogenic capacity. Seventy-nine experimental genes were studied on all samples (assay identification in [17]). Relative expression was calculated using the 2^(−ΔCt)^ method normalising to the reference gene* HPRT*. Level of induction was established using 2^(−ΔΔCt)^.

### 2.5. Telomere Length Measurements

Relative telomere lengths were measured in 20 ng gDNA by qPCR amplification of telomere repeats (T) in relation to a single copy gene, 36B4 (S); the subsequent T : S ratio enabled calculation of telomere length in base pairs (bp) [18]. Reaction components were 2x SYBR Green PCR Master Mix (ThermoFisher) and primers Tel1 (270 nM), Tel2 (900 nM), 36B4u (300 nM), and 36B4d (500 nM) [18] (Eurofins Genomics, Ebersberg, Germany).

### 2.6. Methylation Signature

To track the state of cellular senescence with regard to accrued PD, the level of methylation of six CpG islands was measured in twenty-four gDNA samples (from 7 donors) from across the expansion period according the method of Koch [12] by Cygenia GmbH (Aachen, Germany).

### 2.7. Statistical Analysis

Statistical analysis was performed using IBM SPSS Statistics 21. Normality of age and p0 PD distributions were determined using the Shapiro-Wilk test (*p* = 0.118 and 0.073, resp.). Correlations were defined using Spearman's rho correlations. Paired analysis of gene expression data was carried out with Wilcoxon Signed Rank test as the data was nonparametric and Mann–Whitney was used for comparison of calculated groups. Gradients were determined from Microsoft Excel equation of the line function. Confidence interval figures were prepared using GraphPad Prism 7.02.

## 3. Results

### 3.1. Basic Characterisation of Culture Expanded MSCs

**Figure 1:**
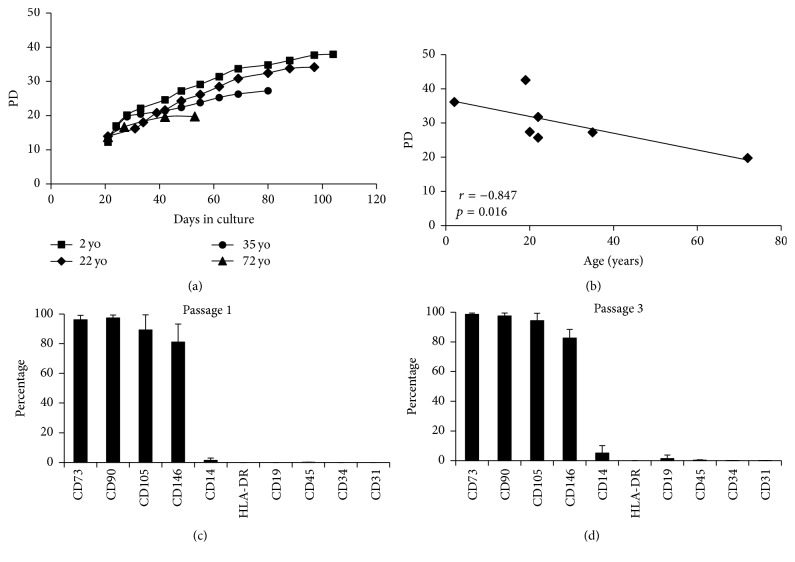
Basic characterisation of culture expanded MSCs. (a) Four donors representing the variable longevity of MSC cultures which is (b) dependent upon donor age (*n* = 7). (c) Confirmation of the ISCT phenotype for MSCs at passage 1 (15.24 PD ± 0.35) and (d) passage 3 (20.23 PD ± 1.4), *n* = 3. Error bars are standard deviation.

Cultivation of MSCs from a widely distributed age range (2–72 years) demonstrated that the longevity of cultures was vastly different and that senescence occurred after a variable number of PDs (Figure 1(a) illustrates 4 of the 7 cultures). The number of PDs that occurred prior to senescence correlated negatively with donor age (Figure 1(b)) [9, 10, 16]. Throughout culture expansion, the cells maintained their MSC phenotype; phenotypic data for p1 and p3 cultures (*n* = 3, corresponding to mean 15.2 and 20.2 PDs, resp.) are shown in Figure 1(c).

Subsequently, and in order to identify markers representative of function and utility rather than individual donor specific trends, data from all donors were pooled and studied collectively.

### 3.2. Osteoprogenitor Capacity of MSCs at Different Stages of Cultivation

**Figure 2:**
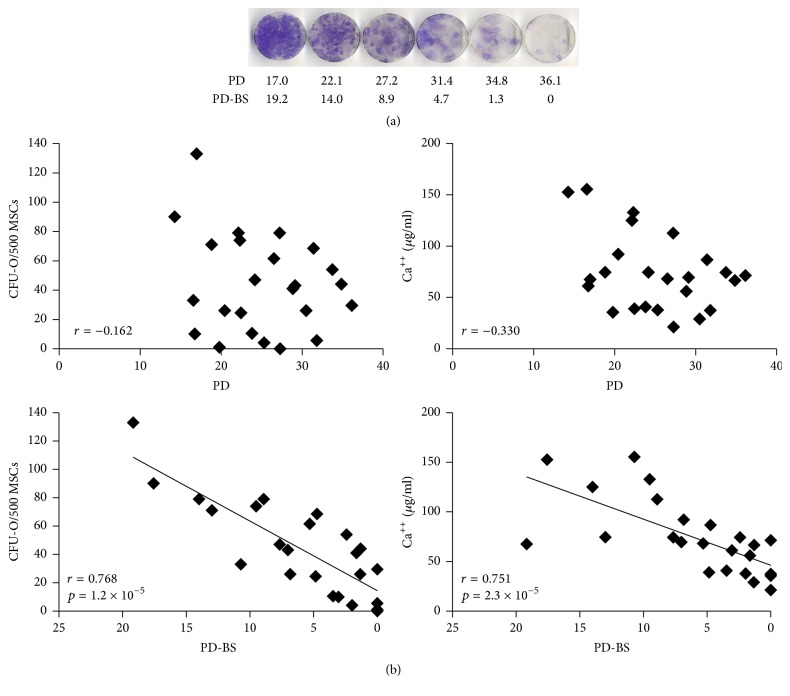
MSC osteogenic capacity in relation to PD and remaining PD, before senescence. (a) Decline in osteogenic differentiation capacity of MSCs from a representative donor (alkaline phosphatase). (b) Functional measures of osteogenesis. PD: population doubling; PD-BS: population doubling before senescence.

Consistent with previous studies [16, 19], gradual loss of osteogenic colonies (CFU-O) was observed in all donor cultures (series from one donor shown in Figure 2(a)). However, using pooled data (representing donor diversity), although CFU-O decline was still observed, it was evident that neither CFU-O nor calcium production had strong relationships with accrued PDs (Figure 2(b) top), that is, with cultures' in vitro age. Much stronger, statistically significant, correlations were found for both CFU-O and Ca^++^ deposition against PD-BS (Figure 2(b) bottom) indicating their dependence on both accrued PDs and donor age (a factor determining cultures' longevity) [9, 10].

Additionally, the upregulation (2^−ΔΔCt^) of the* ALPL *(alkaline phosphatase) gene in response to osteogenic stimulation was also significant in relation to PD-BS (*r* = 0.470, *p* = 0.020), but not PD (*r* = 0.026).

### 3.3. Gene Expression of MSCs at Different Stages of Cultivation

**Figure 3:**
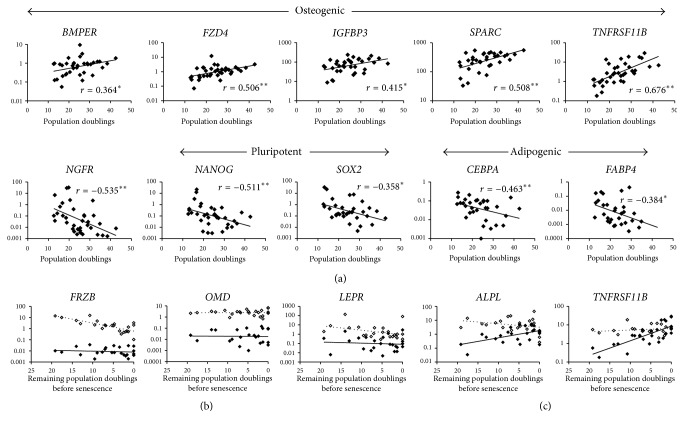
Gene expression changes with time in vitro and effect of osteoinduction. (a) Transcripts with the strongest relationship with PD (36 points from 7 donors). (b) Induced gene expression patterns compared to undifferentiated MSCs throughout culture expansion. ^*∗*^*p* < 0.05;  ^*∗∗*^*p* < 0.01. (b) Upregulated genes; (c) time/passage variable genes; solid diamond/line undifferentiated MSCs; hollow diamond/dotted line osteodifferentiated MSCs.

Initially, gross gene expression changes were measured at passage 0 (mean 14.8 PD) and compared to their presenescent state (passage immediately prior to <1 PD in 1 week; mean 29.9 PD). As might be expected from age-diverse samples the spread of gene expression was often broad and therefore statistical analysis suggested that the RNA message of the majority of the molecules tested (60/79 genes) did not change between these two stages (*p* > 0.05; no change also stated if less than 2-fold change). The genes showing the greatest mean fold increase at presenescence were* TNFRSF11B* (13-fold) and* FZD4* (5-fold) with 13 others exhibiting 2–4-fold increases (Supplementary Figure  1 in Supplementary Material available online at https://doi.org/10.1155/2017/6129596). Two molecules,* NANOG* (84-fold) and* TWIST1* (5-fold), were significantly lower at presenescence and* WIF1* became undetectable in 6/7 samples. As well as being the most upregulated molecule at presenescence,* TNFRSF11B*/osteoprotegerin also had the strongest relationship with accrued PD (followed by* SPARC*/osteonectin (Figure 3(a)). Negative relationships were observed for a markers of stem cell pluripotency [20]:* NANOG* and* SOX2*, MSC marker* NGFR* [21], and Wnt regulators;* SFRP1* and* WIF1*, as well as* TWIST1* and* TWIST2* (not shown) and adipogenesis markers, namely,* CEBPA* and* FABP4* (Figure 3(a)). Chondrogenesis-associated molecules showed no obvious relationship with PD (Supplementary Figure 2). Overall, and regardless of donor age, culture expansion appeared to cause the loss of pluripotency and adipogenic markers whilst cultures' osteogenic commitment increased (evident by increases in mature osteoblast markers such as* SPARC* and* TNFRSF11B*/osteoprotegerin). Combined with functional data on the loss of CFU-O with accrued PDs (Figure 2), these data indicated that late passage cultures comprised poorly proliferative, osteogenically committed cells on their way to senescence.

When considered at all time points, osteogenic stimulation caused the upregulation of several (24) genes, but only four were reduced (Table 1). However, when gene expression was compared before and after osteogenic induction longitudinally in relation to PD-BS, responses fell into 4 categories: (1) upregulated, (2) downregulated, (3) noninducible, and (4) passage/time variable (Figure 3(b) and Supplementary Figure 3). Some molecules were always upregulated, such as* FRZB* and* OMD*, whereas* TNFRSF11B* and* ALPL* became less induced closer to senescence as the basal level itself is increased.

Whilst these data showed that inability to further upregulate* TNFRSF11B* and* ALPL* could be a feature of presenescent MSCs and hence be indicative of culture's remaining lifespan, its value as a predictive test is low, since osteogenic differentiation assays are notably very time consuming. With this in mind, postinduction marker expression was not considered for prediction purposes.

### 3.4. Identification of Gene Transcripts and Other Parameters with Predictive Potential for MSC Culture Longevity

The search for predictive markers was next narrowed to those gene transcripts showing strong correlations with both PDs and PD-BS (Table 2), although association with all four measures (PD, PD-BS, CFU-O, and Ca^++^ deposition) was required for candidature and those with the strongest potential (relationship with PD-BS rho > 0.6) were* BMPER*,* SPARC*, and* TNFRSF11B* (Table 2). None of these were strong enough in isolation to be predictive at 95% confidence (Supplementary Figure 4). In having the steepest gradient in addition to its functional correlations (Table 2)* SPARC* was the leading candidate for use in conjunction with other known MSC replicative ageing-associated factors: telomere measurement [9] and gene methylation markers [12], both of which were investigated independently and for possible inclusion with* SPARC*. Importantly, when tested in a second medium, StemMACS,* SPARC* retained its relationships with PD-BS and PD, whereas* BMPER* and* TNFRSF11B* did not (Supplementary Figure 5). Standard MSC surface markers were not considered for this analysis as they showed no changes with accrued PDs (Figure 1(c)). However, CD markers were comprehensively tested in Siegel et al. [22], where several candidate molecules were identified for consideration in predictive testing.

**Figure 4:**
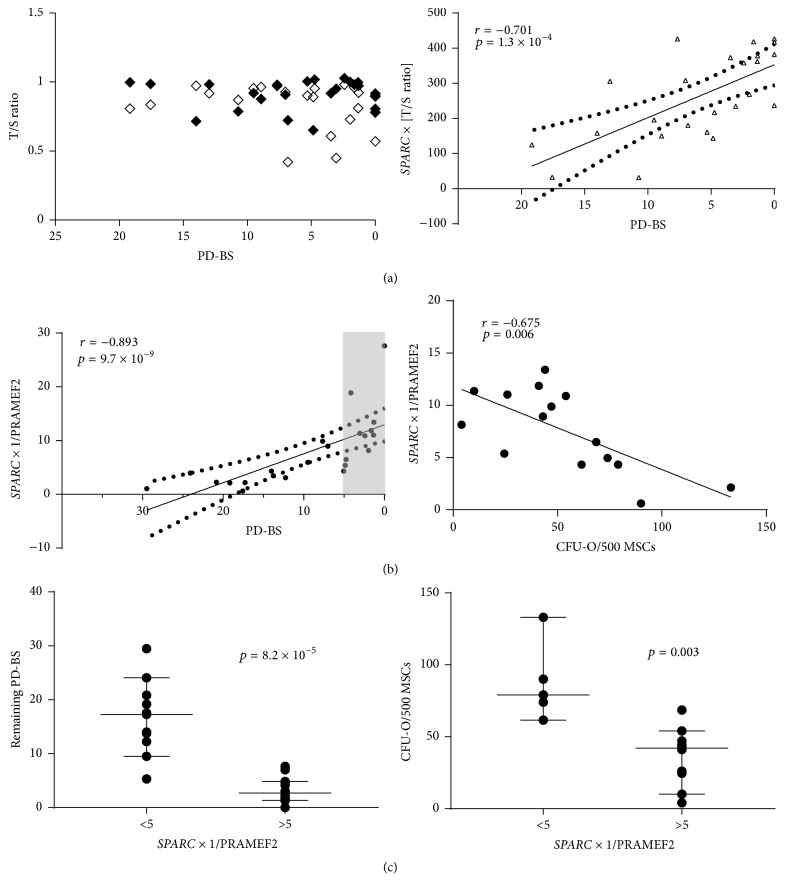
Combination of* SPARC* and other parameters with predictive potential for MSC culture longevity. (a) No relationship between T/S ratio and PD-BS (left; solid: culture expanded, hollow: postosteogenic induction), but correlation between* SPARC* × [T/S ratio] and PD-BS (solid line: linear regression; dotted line: 95% confidence). (b) Left: 95% confidence in calculated values of* SPARC* × [1/PRAMEF2] in relation to PB-BS (shaded area ≤5 PD-BS); right: relationship between* SPARC* × [1/PRAMEF2] and CFU-O (c). Prediction model of calculation values <5 being indicative of higher remaining PD-BS (left) and osteogenic potential (right). Whisker range 2.5%–97.5% confidence, line at median.

As expected for this age-diverse cohort, no significant relationship was found between the telomere lengths and PD or PD-BS (latter shown in Figure 4(a) left). However, the process of clonal differentiation itself lowered the T/S ratio (the relative measure of telomere to single copy gene) [23] which equated to a reduction in telomere length by a median value of 94 bp (23 points from 4 donors, *p* = 0.038) which may be representative of the fact that colony-forming cells were plated at a low density and were allowed to proliferate before being placed in osteogenic conditions. When combined with* SPARC *(*SPARC* × [undifferentiated T/S ratio]), a correlation was observed in relation to PD-BS; however the majority of the points (63%) fell outside of the 95% confidence limits (Figure 4(a) right); therefore application of T/S ratios was not pursued further.

Another potential marker of MSC replicative ageing, DNA methylation, was independently investigated (blind) with regard to MSCs' predicted passage number (PPN). The PPN was deduced by testing six CpG islands [12] and correlated strongly with actual cumulative/accrued PD and PD-BS (*r* = 0.949, *p* = 1 × 10^−14^ and *r* = −0.788, *p* = 2 × 10^−6^, resp., data not shown). When the individual CpG islands were studied, their methylation levels remained more closely correlated with PD (Table 3); however positive associations with PD-BS for PRAMEF2, SELP, KRTAP13-3, and CASP14 were observed. None of the CpG islands showed any significant relationship with osteogenic capacity (CFU-O or Ca^++^ deposition).

Utilizing the strength of the CpG island correlations with PD and PD-BS (Table 3) and relative levels of* SPARC* mRNA with osteogenic capacity, PD, and PD-BS (Table 2), calculations incorporating these values were also tested for more robust predictability. The relative level of* SPARC* was multiplied by methylation value for islands with positive associations with PD (CASR and GRM7) or reciprocal value for negative associations (PRAMEF2, SELP, CASP14, and KRTAP13-3). Correlations were observed for all tests for both PD (*r* ≥ 0.589, *p* ≤ 0.003) and PD-BS (*r* ≥ 0.794, *p* ≤ 6 × 10^−6^); however only PD-BS was considered further for predictive capability. With more than 5 remaining PD-BS, all points (except one) fell within the 95% confidence limits for* SPARC* × [1/PRAMEF2] (Figure 4(b)). This calculation additionally had the strongest correlation with CFU-O (Figure 4(b)) although it is notable that all* SPARC*/CpG relationships correlated with CFU-O (*r* > −0.532, *p* < 0.05). Using an arbitrary calculation value for* SPARC *× [1/PRAMEF2] of ≤5 against calculated trend lines (*y* = 0.0778*x* + 11.683),* SPARC* × [1/PRAMEF2] yielded remaining ≥15 PD-BS, and ≥86 CFU-O/500 MSCs. Even when reduced to 95% confidence this value (*SPARC* × [1/PRAMEF2] ≥ 5) could predict ≥10 PD-BS (Figure 4(c) left) and ≥62 CFU-O/500 MSCs (Figure 4(c) right). If the calculated value was greater than five then there would be no guarantee (95% confidence limit) of more than 5 PDs remaining before senescence. The differences in the above or below five categories were highly significant (Figure 4(c)); therefore the calculation could be considered reliable and could be applied early in MSC culture expansion.

## 4. Discussion

Since MSCs have great therapeutic potential for tissue rescue and repair [24], the ability to predict the utility and potency of a BM-MSC sample prior to or at an early stage of culture expansion would be invaluable. For bone repair applications, and particularly for large bone defect reconstruction, the cells' ability to proliferate and populate the void prior to differentiation is paramount; indeed >1500 cells per cm^3^ are required for bone union according to Hernigou et al. [25]. Some predictions about MSC proliferation lifespan can be made with regard to MSC donor age [9, 10] but donor age alone is likely to be a very poor predictor as some studies reported no clear association [26] and it does not take into account a culture's in vitro proliferative history. Molecular markers indicative of MSC replicative ageing have been investigated previously [6, 22, 27, 28]; however in this study efforts were made to combine these two essential factors and to develop a predictive test of the culture's remaining proliferative capacity applicable to a broad donor age range and independent of its previous replicative history.

The MSCs used in this study represented a broad biological donor age range (70 years) testing both young and old extremes in an exaggerated model investigating molecular markers throughout culture expansion up to senescence thus allowing putative markers to be thoroughly scrutinized. All cultures had a typical MSC phenotype and their lifespans to senescence correlated with donor age, as expected [8–10]. Many MSC-related gene transcripts [17, 29, 30] showed no significant changes from p0 to senescence; this was not unexpected given the magnitude of change that has already occurred from the native (uncultured) state to early passage MSCs [17, 31]. Though the cohort size was small, the range of proliferation, time (days) and PD, observed with respect to donor age agreed with previous studies relating to human MSCs [8–10]. That* NANOG*,* TWIST1*, and* WIF1* declined during expansion was not unexpected since all have been subjects of increased proliferation studies [32–34] which is indicative of the known loss of MSC multipotency during extended cultivation [35, 36].

When the behaviour osteogenesis-related and other genes were investigated in relation to MSC replicative ageing status (measured by PDs), an increased expression of many genes was found. This strongly supported the notion that MSCs progressively commit to osteogenic lineage during prolonged cultivation [7, 37] parallel with the loss of multipotentiality. Interestingly, this was further supported by genes such as* TNFRSF11B* and* ALPL* no longer needing to be upregulated closer to senescence as the uninduced levels matched the osteoinduced levels of the younger cultures/earlier passages. Conversely, the relative expression levels of* CEBPA* and* FABP4* steadily declined indicating the loss of adipogenic potential.

Throughout the cultivation period clonal osteoprogenitor assays were performed and similar to our previous study showed a gradual loss of CFU-O with increased passaging [16]. However, both CFU-O numbers and calcium deposition assays showed stronger correlations with PD-BS compared to PD. This indicated that MSC osteogenic potential was more related to remaining proliferation capacity (also determined by donor age), than simply a culture's time in vitro [7, 38].

Of the genes studied,* SPARC* was the best candidate marker due to its functional correlations and additionally its superior expression range (between early and late passage cultures); however it could not provide 95% confidence for predicting PD-BS alone. This leads to modelling with other parameters: telomere and methylation markers. In spite of literature supporting the loss of telomere length being a feature of MSC replicative ageing [9, 10, 16] no correlation was found in either culture expanded MSCs or osteodifferentiated MSCs, although the differentiated MSCs did have significantly shorter telomeres. Modelling calculations in conjunction with* SPARC* were attempted but were not predictive.

The methylation of CpG islands [12] collectively had very strong associations with PD, which remained for PD-BS albeit reduced; however for predictability PRAMEF2 provided the best indicator of remaining PD-BS when in conjunction with* SPARC* (*SPARC* × [1/PRAMEF2]). PRAMEF2 currently has no known function and is one of several paralogous duplications of PRAMEF1 located on chromosome 1p36.21 [39]. It is arguable that the 2- and 72-year-old donors may bias this study; however we further tested this model without data from these donors', and even with less data/lower power the correlations and testing of the model remained strong for* SPARC* × [1/PRAMEF2] (Supplementary Figure 6), thus reinforcing the utility of this calculation.

We acknowledge that in this study MSC culture expansion was primarily performed with the addition of FGF2, an additive used for clinical-grade MSC manufacture [13] and known to maintain MSC stemness by inhibiting cellular senescence and by promoting proliferation [40] but not to affect MSC proliferative lifespan [19]. The number of cultures tested was comparable [41] or even higher than used in previous predictive modelling studies [29] and uniquely a broad age-range feature was incorporated into the study. In vivo segmental bone defect animal model investigations will be ultimately needed to prove the translational value of the proposed predictive formula prior to its application to human clinical studies.

## 5. Conclusions

Since both proliferative and osteogenic capacity of MSCs are important for bone tissue repair then it was essential to understand how both factors relate to each other during MSC cultivation. This study confirmed that MSC cultivation leads to a pronounced loss of the multipotency and adipogenic lineage gene expression in BM MSCs. The fact that the loss of osteoprogenitors correlated more with the remaining culture's lifespan than its time in culture suggests that donor age influence cannot be ignored when culture effects on osteogenesis are considered. This study developed a predictive test of MSC culture longevity and associated osteogenic capacity, irrespective of donor age. Similar to other predictive assays proposed in the past [12, 29], this assay employs a formula based on a combination of two parameters, in our case gene expression level for* SPARC* and PRAMEF2. It capitalises on the greatest expression range of* SPARC* across time in culture, coupled with highly accurate CpG island PRAMEF2. The fact that* SPARC* is unchanged between the native CD271-positive BM MSCs and early passage MSCs [17] suggests that this may be a viable test even before MSC culturing.

## Supplementary Material

The supplementary document contains evidence of (1) the upregulation of genes between passage 0 and senescence, (2) the effect of culture expansion upon chondrogenic genes and (3) the effect of osteogenic induction on selected genes. Furthermore, in support of developing the predictive model, the evidence for selection of the candidate molecules is shown (4), with their robustness in alternative medium (5). The predictive model was further tested excluding the more diverse-in-age donors (6).

## Figures and Tables

**Table 1:** Genes upregulated following osteogenic induction.

Gene	Fold upregulation	Significance
	(all time points)	
*FRZB*	256.9	*∗∗∗∗*
*OMD*	72.4	*∗∗∗∗*
*PDGFRL*	55.7	*∗∗∗∗*
*LEPR*	29.2	*∗∗∗∗*
*IGF2*	22.6	*∗∗∗∗*
*NGFR*	19.6	*∗*
*MSX1*	10.4	*∗∗*
*ANGPTL4*	8.2	*∗∗∗∗*
*MT2A*	7.7	*∗∗∗∗*
*SFRP1*	7.3	*∗∗∗∗*
*TGFBR2*	5.7	*∗∗∗∗*
*TGFBR3*	5.7	*∗∗∗∗*
*SPP1*	5.5	*∗∗∗∗*
*ALPL*	4.9	*∗∗∗∗*
*SORT1*	4.4	*∗∗∗∗*
*DVL2*	3.2	*∗∗∗∗*
*PDGFRA*	3.2	*∗∗∗∗*
*BMPER*	2.8	*∗∗*
*S1PR1*	2.8	*∗∗∗∗*
*FZD1*	2.7	*∗∗∗∗*
*CSPG4*	2.6	*∗∗∗∗*
*FZD4*	2.5	*∗∗∗∗*
*NGFRAP1*	2.4	*∗∗∗*
*FGFR1*	2.1	*∗∗∗∗*
*SOX9*	0.5	*∗∗*
*NES*	0.4	*∗∗*
*VEGFA*	0.3	*∗∗∗∗*
*SFRP4*	0.2	*∗∗*

Only genes with significant >2-fold induction: ^*∗*^*p* < 0.05, ^*∗∗*^*p* < 0.01, ^*∗∗∗*^*p* < 0.001, and ^*∗∗∗∗*^*p* < 0.0001.

**Table 2:** Transcript relationships with PD-BS and osteoprogenitor indicators.

Gene	Correlation with PD-BS (rho = (*p*))	Gradient	Range (min–max)	Also related to PD?	Relationship with CFU-O?	Relationship with Ca^++^?
Higher expression closer to senescence						
* ACVR2A*	−0.454^*∗∗*^	−0.0727	0.07–3.41	Y	Y	Y
* ALPL*	−0.348^*∗*^	−0.1095	0.03–5.26		Y	Y
* ANGPTL4*	−0.422^*∗*^	−0.0336	0.01–1.57		Y	Y
* BAMBI*	−0.454^*∗∗*^	−0.2279	0.29–8.67	Y	Y	Y
* BMPER*	−0.686^*∗∗*^	−0.0985	0.06–9.28	Y	Y	Y
* COL1A1*	−0.551^*∗∗*^	−7.2721	6.35–302	Y	Y	Y
* COL1A2*	−0.340^*∗*^	−10.771	14.6–632			
* FGFR1*	−0.453^*∗∗*^	−0.2315	0.61–13.6	Y		Y
* FZD4*	−0.477^*∗∗*^	−0.0789	0.07–12.2		Y	Y
* IGFBP3*	−0.417^*∗*^	−5.1189	8.78–236	Y		Y
* JAG1*	−0.465^*∗∗*^	−0.1876	0.25–27.0			Y
* SFRP4*	−0.511^*∗∗*^	−0.1091	0.004–35.4	Y	Y	Y
*SORT1*	−0.331^*∗*^	−0.1515	0.134–5.68		Y	Y
^#^*SPARC*	−0.753^*∗*^	−16.614	33.2–545	Y	Y	Y
* TNFRSF11B*	−0.642^*∗∗*^	−0.7225	0.179–29.2	Y	Y	Y
Lower expression closer to senescence						
* CEBPA*	0.358^*∗*^	0.0012	0.001–0.119	Y		
* SFRP1*	0.369^*∗*^	0.0034	0.002–0.634	Y		
* SOX2*	0.390^*∗*^	0.3751	0.005–22.9	Y		

^*∗*^ *p* < 0.05; ^*∗∗*^*p* < 0.01; Y (yes) = *p* < 0.05; empty = no significance. ^#^Strongest candidate for prediction model based on multiple correlations and steepest gradient.

**Table 3:** Correlations of methylation levels of CpG islands in relation to culture expansion.

CpG island	PD	PD-BS
*GRM7*	0.606^*∗∗*^	−0.567^*∗∗*^
*CASR*	0.527^*∗∗*^	−0.640^*∗∗*^
*PRAMEF2*	−0.946^*∗∗*^	0.775^*∗∗*^
*SELP*	−0.934^*∗∗*^	0.737^*∗∗*^
*CASP14*	−0.916^*∗∗*^	0.640^*∗∗*^
*KRTAP13-3*	−0.941^*∗∗*^	0.661^*∗∗*^

^*∗∗*^ *p* < 0.01.
