# Supplementary material for: Predicting the Remaining Lifespan and Cultivation-Related Loss of Osteogenic Capacity of Bone Marrow Multipotential Stromal Cells Applicable across a Broad Donor Age Range
Source: Stem Cells Int. 2017 Feb 19;2017:6129596. doi: 10.1155/2017/6129596 (PMC5337353; doi:10.1155/2017/6129596)
Supplement: Supplementary file 1 — The supplementary document contains evidence of (1) the upregulation of genes between passage 0 and senescence, (2) the effect of culture expansion upon chondrogenic genes and (3) the effect of osteogenic induction on selected genes. Furthermore, in support of developing the predictive model, the evidence for selection of the candidate molecules is shown (4), with their robustness in alternative medium (5). The predictive model was further tested excluding the more diverse-in-age donors (6). [file 6129596.f1.docx]

Supplementary Document

# Title: Predicting the remaining lifespan and cultivation-related loss of osteogenic capacity of bone marrow multipotential stromal cells irrespective of donor age

**Authors: Sarah M Churchman^1,2^, Sally A Boxall^1,3^, Dennis McGonagle^1,2^, Elena A Jones^1,2*^**

^1^Leeds Institute of Rheumatic and Musculoskeletal Medicine, University of Leeds, Beckett Street, Leeds, LS9 7TF, UK.

^2^NIHR-Leeds Musculoskeletal and Biomedical Research Institute, Chapel Allerton Hospital, Chapeltown Road, Leeds Teaching Hospital NHS Trust, Leeds, LS7 4SA, UK

^3^School of Molecular and Cellular Biology, Faculty of Biological Sciences, University of Leeds, Leeds, LS2 9JT

*Corresponding Author: msjej@leeds.ac.uk

Contents:

**Supplementary Figure 1.** Gene transcripts significantly upregulated between p0 and pre-senescent passage in n=7 age-diverse donors.

**Supplementary Figure 2.** Effect of culture expansion on chondrogenesis-associated gene transcripts.

**Supplementary Figure 3.** Examples of downregulated (A) and non-inducible (B) gene transcripts, following osteogenic induction.

**Supplementary Figure 4.** Relationship and confidence levels of candidate molecules with PD-BS.

**Supplementary Figure 5.** Relationship of candidate molecules with PD and PD-BS in MSCs expanded in StemMACS medium.

**Supplementary Figure 6.** Testing of *SPARC* x [1/PRAMEF2] model following exclusion of 2 and 72 year old donor data.

**
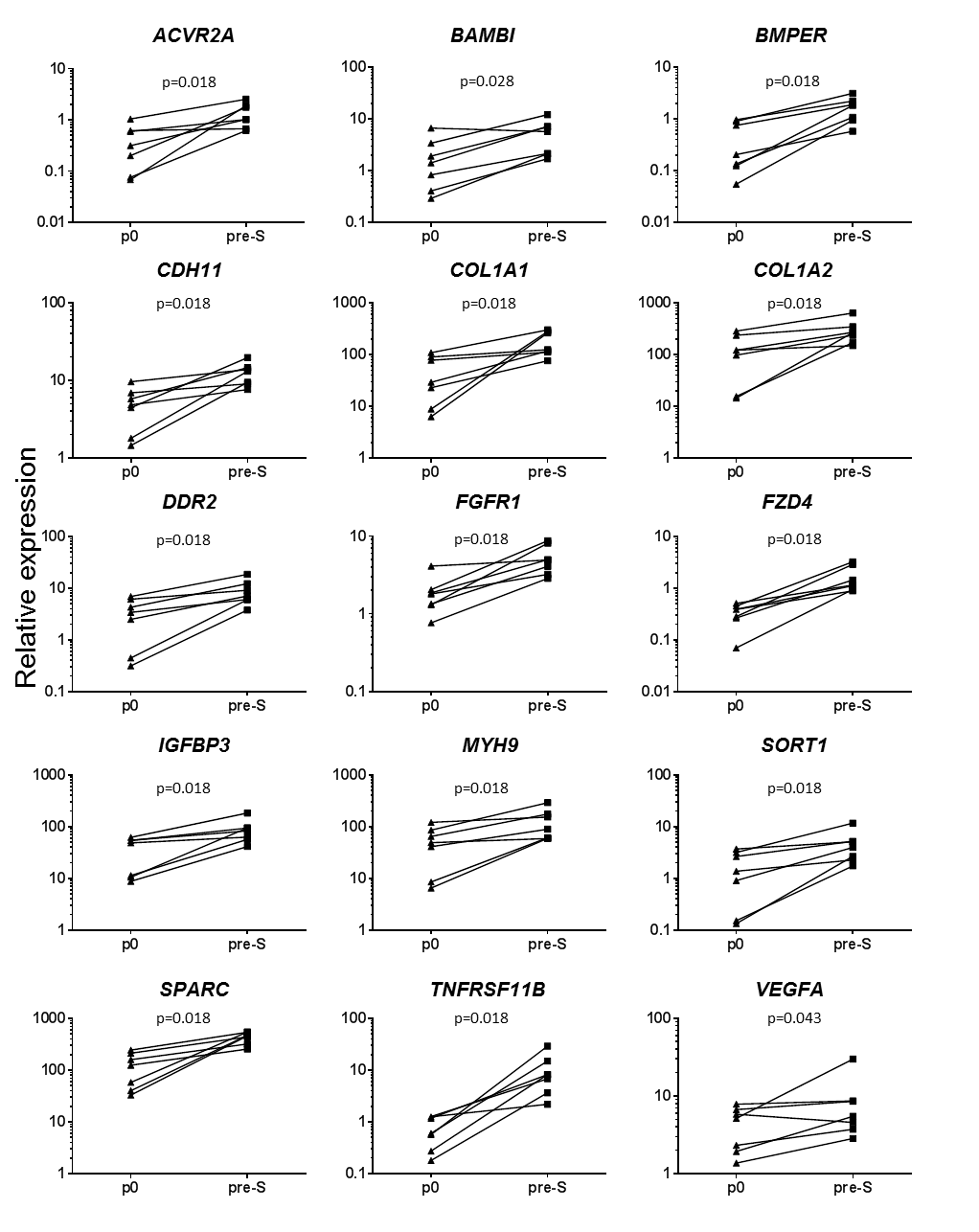
**

**Supplementary Figure 1. Gene transcripts significantly upregulated between p0 and pre-senescent passage in n=7 age-diverse donors.** Includes 13 gene transcripts exhibiting 2-4-fold increases between early passage (p0) and their pre-senscent passage (pre-S), as well as *TNFRSF11B* and *FZD4* (13- and 5- fold, respectively).


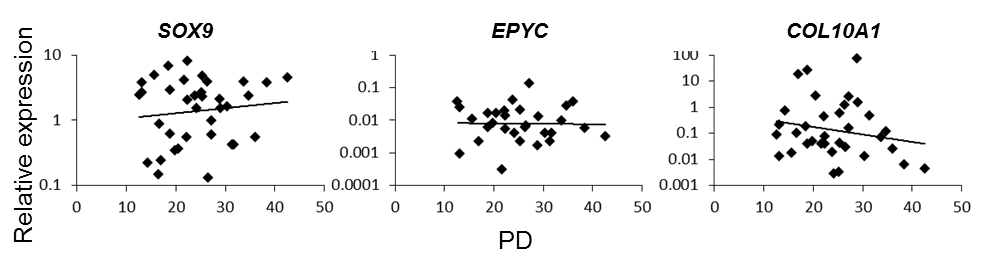


**Supplementary Figure 2. Effect of culture expansion on chondrogenesis-associated gene transcripts.** PD = population doubling.


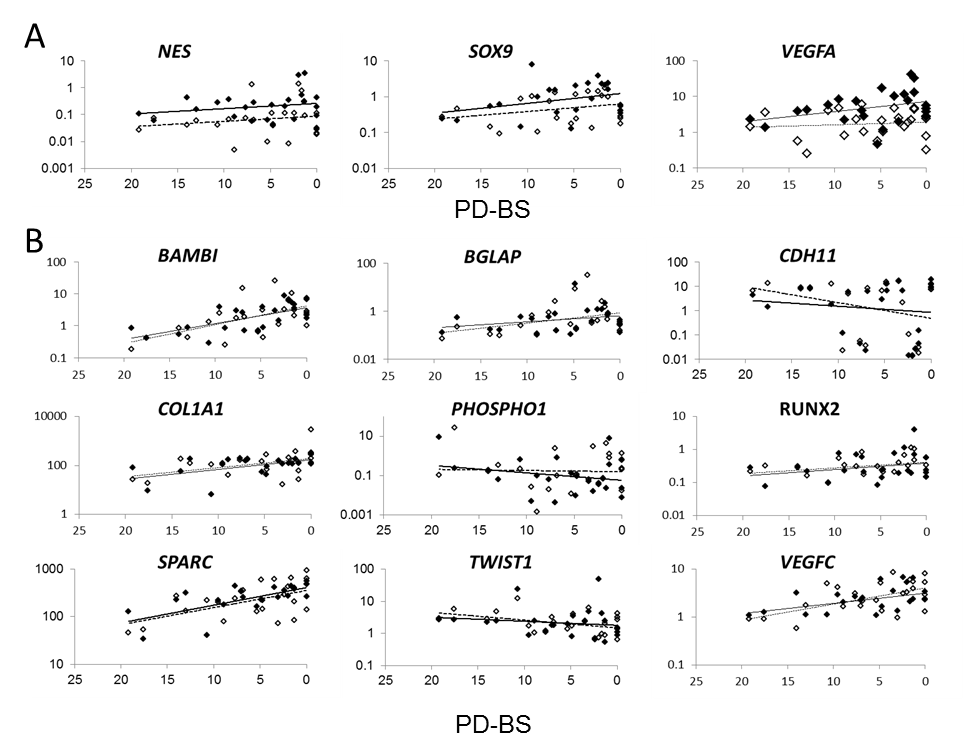


**Supplementary Figure 3.** **Examples of downregulated (A) and non-inducible (B) gene transcripts, following osteogenic induction.** Closed diamonds/solid line = undifferentiated, open diamonds/dotted line = differentiated. PD-BS – population doublings before senescence.


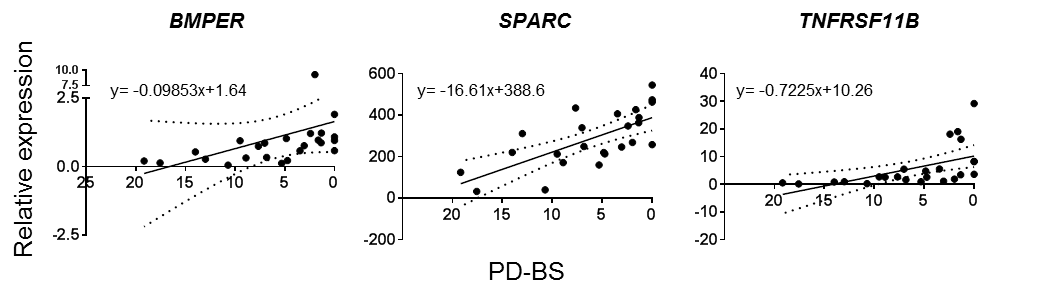
**Supplementary Figure 4. Relationship and confidence levels of candidate molecules with PD-BS.** Solid linear regression (solid line) and equation shown on chart, dotted lines = 95% confidence. PD-BS – population doublings before senescence.

**
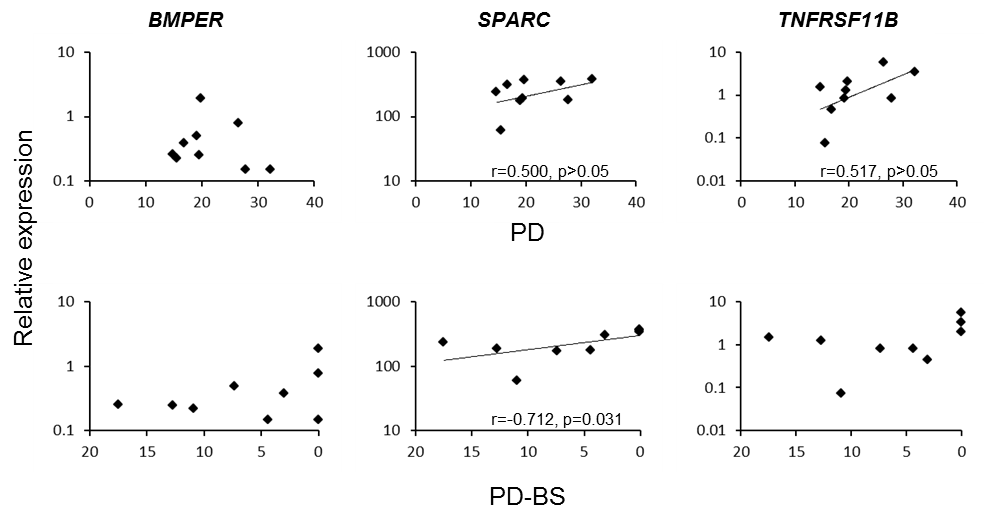
**

**Supplementary Figure 5. Relationship of candidate molecules with PD and PD-BS in MSCs expanded in StemMACS medium.** PD - population doublings, PD-BS –PD-before senescence. Correlation reported above r=0.5.

**
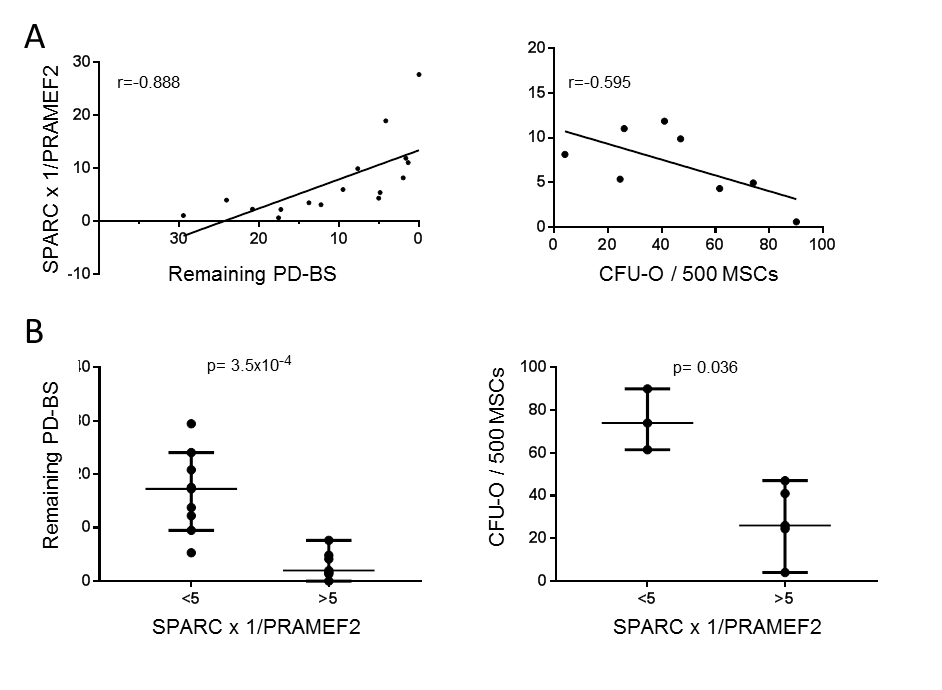
**

**Supplementary Figure 6. Testing of *SPARC* x [1/PRAMEF2] model following exclusion of 2 and 72 year old donor data.** A, Correlation of calculation value with remaining PD-BS and CFU-O/500 MSCs and B, testing and validation of the >5< hypothesis. PD-BS – population doublings before senescence.
